# Oblique Lateral Interbody Fusion versus Transforaminal Lumbar Interbody Fusion in Degenerative Lumbar Spondylolisthesis: A Single-Center Retrospective Comparative Study

**DOI:** 10.1155/2021/6693446

**Published:** 2021-03-20

**Authors:** Xing Du, Yuxiao She, Yunsheng Ou, Yong Zhu, Wei Luo, Dianming Jiang

**Affiliations:** Department of Orthopedics, The First Affiliated Hospital of Chongqing Medical University, 400016 Chongqing, China

## Abstract

**Objective:**

To compare the efficacy of oblique lateral interbody fusion (OLIF) and transforaminal lumbar interbody fusion (TLIF) in single-level degenerative lumbar spondylolisthesis (DLS).

**Methods:**

A retrospective analysis of patients who underwent single-level DLS surgery in our department from 2015 to 2018 was performed. According to the surgical method, the enrolled patients were divided into two groups, namely, the OLIF group who underwent OLIF combined with percutaneous pedicle screw fixation (PPSF) and the TLIF group. Clinical outcomes included operation time, operation blood loss, postoperative drainage, hospital stay, visual analog scale (VAS) score, Oswestry disability index (ODI), and complications, and imaging outcomes included upper vertebral slip, intervertebral space height (ISH), intervertebral foramen height (IFH), intervertebral space angle (ISA), lumbar lordosis (LL), and bone fusion rate. All outcomes were recorded and analyzed.

**Results:**

A total of 65 patients were finally included, and there were 28 patients and 37 patients in the OLIF group and the TLIF group, respectively. The OLIF group showed shorter operation time, less blood loss, less postoperative drainage, and shorter hospital stay than the TLIF group (*P* < 0.05). The ISH, IFH, ISA, and LL were all larger in the OLIF group at postoperative and last follow-up (*P* < 0.05), but the degree of upper vertebral slip was found no difference between the two groups (*P* > 0.05). The bone graft fusion rate of OLIF group and TLIF group at 3 months, 6 months, and last follow-up was 78.57%, 92.86%, and 100% and 70.27%, 86.49%, and 97.30%, respectively, and no significant differences were found (*P* > 0.05). Compared with the TLIF group, the OLIF group showed a superior improvement in VAS and ODI at 1 month, 3 months, and 6 months postoperative (*P* < 0.05), but no differences were found at 12 months postoperative and the last follow-up (*P* > 0.05). There was no significant difference in complications between the two groups, with 4 patients and 6 patients in the OLIF group and TLIF group, respectively (*P* > 0.05).

**Conclusions:**

Compared with TLIF, OLIF showed the advantages of less surgical invasion, better decompression effect, and faster postoperative recovery in single-level DLS surgery.

## 1. Introduction

Lumbar spondylolisthesis is defined as the forward slip of the upper vertebrae relative to the lower. There are many causes of lumbar spondylolisthesis, including degeneration, trauma, dysplasia, and pathology, among which degenerative lumbar spondylolisthesis (DLS) is the most common [1]. It is reported that the incidence of DLS is about 5% to 7% and often causes low back pain, lower limb pain, or weakness, and even cauda equina syndrome in severe cases [2]. The treatment of DLS mainly includes conservative and surgical treatment. For patients with no obvious efficacy after regular conservative treatment for more than 3 months, surgery should be considered [3].

The surgical methods of DLS mainly include anterior and posterior surgery [4]. In anterior lumbar interbody fusion (ALIF), the discectomy and spondylolisthesis reduction can be performed under direct vision, and the correction and maintain of intervertebral space height and lumbar lordosis (LL) can be also achieved by implanting a large cage [5]. But ALIF has a high risk of abdominal vascular or viscera injury, as well as the rate of postoperative complications [6]. In recent years, transforaminal lumbar interbody fusion (TLIF) has been the most commonly used posterior surgery for DLS [7]. By resecting the facet joints, loosening surrounding fibrous tissue, and implanting pedicle screws and intervertebral cage, TLIF can obtain a good spondylolisthesis reduction effect and maintain satisfactory spinal stability [8]. However, patients undergoing TLIF often suffer from chronic back pain after surgery, which may be due to the resection of paravertebral muscle and facet joints [9]. In addition, surgeons need to open the spinal canal in TLIF surgery, and the intraoperative nerve stimulation may also cause numbness or weakness of the lower limbs after surgery [10]. Thus, more and more surgeons have been seeking minimally invasive surgical methods for DLS.

Oblique lateral interbody fusion (OLIF) is a minimally invasive anterior retroperitoneal approach surgery, which has been very popular in recent years. In OLIF surgery, the surgeon enters the retroperitoneal space through blunt separation, pulls the psoas muscle backward, reaches the operative segment or intervertebral space through the anatomical space between the abdominal aorta and the psoas muscle, and performs decompression and fusion procedures [11]. OLIF was reported with satisfactory efficacy in lumbar degenerative diseases in cohort studies [12, 13]; however, no study compared its efficacy with TLIF surgery in DLS. Our previous study conclude that OLIF combined with percutaneous pedicle screw fixation (PPSF) had less surgical trauma and faster pain relief and rapid lumbar function recovery in lumbar tuberculosis [14]. Thus, we really wondered whether OLIF may or may not be extrapolated to the DLS surgery.

Therefore, we conducted this retrospective study to compare the efficacy of OLIF and TLIF in single-level DLS, in order to provide evidence for the application of OLIF in DLS patients.

## 2. Materials and Methods

The ethical approval of this study was obtained from the Ethics Committee of the First Affiliated Hospital of Chongqing Medical University (No. 2020-049), and all the participants gave the informed consent before taking part. This study had been registered in the Chinese Clinical Trial Registry (ChiCTR2000039446). This study was reported according to the STROCSS criteria [15].

### 2.1. Patients

A retrospective analysis of medical records of DLS patients hospitalized in our hospital from 2015 to 2018 was conducted.

*Inclusion criteria:* (1) single-level DLS (L2/3-L4/5). (2) Mild symptomatic DLS (Meyerding Grade: I or II). (3) Age > 18 years. (3) Underwent OLIF combined with PPSF (OLIF group) or TLIF (TLIF group). (4) More than 12 months of follow-up time. (5) Clinical and imaging data were completed.

*Exclusion criteria:* (1) a previous lumbar spinal surgery history. (2) Recurrent DLS after surgical treatment. (3) DLS with severe cardiovascular disease or malignant tumor, etc.

### 2.2. Preoperative Management

X-rays, CT, and MRI were taken in all patients to assess the surgical window between the psoas and abdominal aorta, as well as the extent of upper vertebral slip, spinal canal stenosis, and nerve root compression. Surgery was performed when basic diseases such as diabetes, coronary heart disease, and high blood pressure were under control.

### 2.3. Surgical Procedure

The choice of surgical method was mainly based on the following principles: OLIF combined PPSF was mainly used for patients whose preoperative MRI or CT showed an appropriate operative window between the psoas and abdominal aorta. If preoperative MRI or CT showed no operative window between the psoas and abdominal aorta or the operative window was narrow, TLIF should be considered.

#### 2.3.1. OLIF Group

Place the patient in a lateral supine position after general anaesthesia and use C-arm X-rays to identify the surgical level. Then cut a 4 cm incision in the outer abdominal area, separate the layers of abdominal muscles, and push away the extraperitoneal fat with fingers. Find the front edge of the psoas with a Cobb periosteal stripper, push back the psoas and place an OLIF retractor. After exposing the vertebra, insert a positioning needle and confirm the surgical level by C-arm X-rays and place different extenders to extend the channel to 22 mm. Then, remove the intervertebral disc completely and implant a cage (Medtronic, USA) filled with granular bone, which was derived from allogeneic bone (Gold Bone Way, China). Not any bone fusion promoting substance was used in bone graft materials. Then, place a drainage tube and close the incision layer by layer. Adjust the patient to a prone position and do posterior internal fixation with percutaneous pedicle screw instrumentation (IRENE, China). Finally, use a C-arm X-ray to confirm the good position of posterior fixation and close the posterior incision.

#### 2.3.2. TLIF Group

Place the patient in a prone position after general anaesthesia. Make a posterior median incision after identification of the surgical level by C-arm X-rays. According to preoperative clinical features, the side with lower limb symptoms was defined as the decompression side. Strip the sacrospinous muscle of the decompression side, expose the lamina and facet joints of the surgical level, and then implant the pedicle screws. For the contralateral side, expose the facet joints and implant the pedicle screws via the Wiltes approach. Then, resect part of facet joints and lamina of the decompression side and remove the intervertebral disc completely and implant a cage (Guona, China) filled with granular bone through the intervertebral foramen. The used granular bone was derived from the lamina, spinous process, and facet articular process. However, in most cases, the autologous bone volume was not enough, so they were often mixed with some allogeneic granular bone (Gold Bone Way, China). Not any bone fusion promoting substance was used in bone graft materials. Then, correct the spondylolisthesis by properly pulling up and pressurizing the posterior screw system (IRENE, China) and obtain an appropriate LL by using a prebending rod. After the confirmation of correction by C-arm X-rays, the surgical wound was rinsed and hemostasis was carefully performed. The posterior screw system was then properly pulled up and pressurized in order to correct the spondylolisthesis and obtain an appropriate LL, and a C-arm X-ray was used to confirm the correction. The surgical wound was rinsed, and hemostasis was carefully performed. Finally, place a drainage tube and close the incision layer by layer.

### 2.4. Postoperative Management

In the first 3 days after surgery, antibiotics were used to prevent infection. When postoperative drainage was less than 40 ml/d, the drainage tube was removed. An X-ray examination of the lumbar spine was taken after extubation. After discharge, a modeled rigid lumbar brace was applied continuously for 3 months. Patients were requested to wear the lumbar braces every day when getting out of bed, moving, or sitting. Their family members were asked to supervise the brace wearing, and the medical team conducted telephone follow-up once a week to timely evaluate the patient's compliance. Follow-up of X-rays, CT, and MRI (if necessary) was conducted for 1, 3, 6, and 12 months after surgery.

### 2.5. Outcomes

*Clinical outcomes:* (1) operation time, operation blood loss, postoperative drainage, and hospital stay. (2) Visual analog scale (VAS) score and Oswestry disability index (ODI) at 1, 3, 6, and 12 months postoperative and the last follow-up. (3) Complications.

**Figure 1:**
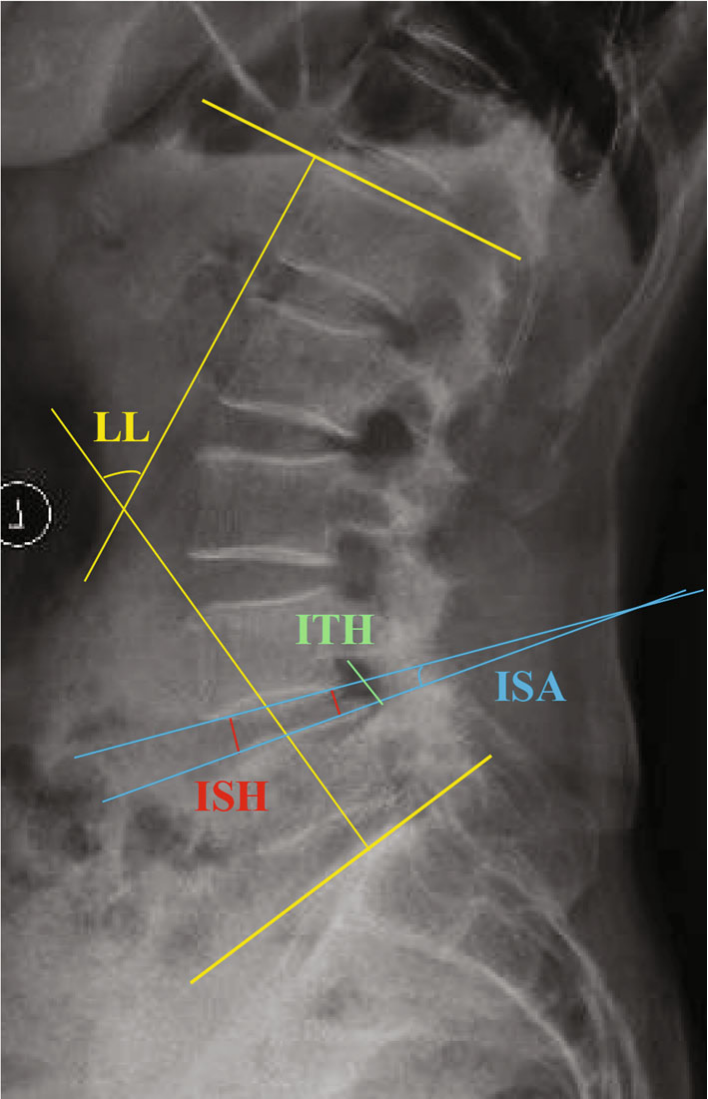
Diagram of the measurement of ISH (red line), ITH (green line), ISA (blue line), and LL (yellow line).

*Imaging outcomes:* (1) the degree of upper vertebral slip: the ratio of the slip distance of the upper vertebrae to the length of the upper endplate of the lower vertebrae. (2) Intervertebral space height (ISH): the mean of anterior and posterior ISH. (3) Intervertebral space foramen (IFH): the distance between the lower margin of the superior pedicle and vertebral body connection and the upper margin of the inferior pedicle and vertebral body connection. (4) Intervertebral space angle (ISA): the angle between the upper and lower endplate of the intervertebral space. (5) Lumbar lordosis (LL): the angle between the upper endplate of the L1 vertebral body and the upper endplate of the S1 vertebral body. The measurement methods of ISH, IFH, ISA, and LL were shown in Figure 1. (6) Bone graft fusion: according to Bridwell et al.'s study [16], bone graft fusion was divided into four levels. Grade I: fused with remodeling and trabeculae. Grade II: graft intact, not fully remodeled and incorporated though; no lucencies. Grade III: graft intact, but a definite lucency at the top or bottom of the graft. Grade IV: definitely not fused with resorption of bone graft and with collapse. Grade I and II were defined as bone graft fusion in this study.

### 2.6. Statistical Analysis

Quantitative data was represented in mean ± standard deviation (SD). Intergroup and intragroup comparison of quantitative data were performed by student *t*-test and paired *t*-test, respectively. The *X*^2^ test and the Mann–Whitney rank-sum test were used for comparison of disordered and ordered qualitative data, respectively. Statistical analysis was conducted by SPSS 19.0 software, and *P* < 0.05 was regarded as a significant difference.

## 3. Results

According to the inclusion and exclusion criteria, our study finally included 65 patients, and there were 28 patients and 37 patients in the OLIF group and the TLIF group, respectively. There were no significant differences in age (*P* = 0.641), gender (*P* = 0.683), body mass index (BMI) (*P* = 0.591), ASA grade (*P* = 0.779), operative level (*P* = 0.890), bone mineral density (BMD) (*P* = 0.101), and follow-up time (*P* = 0.282) between the two groups. (Table 1).

**Figure 2:**
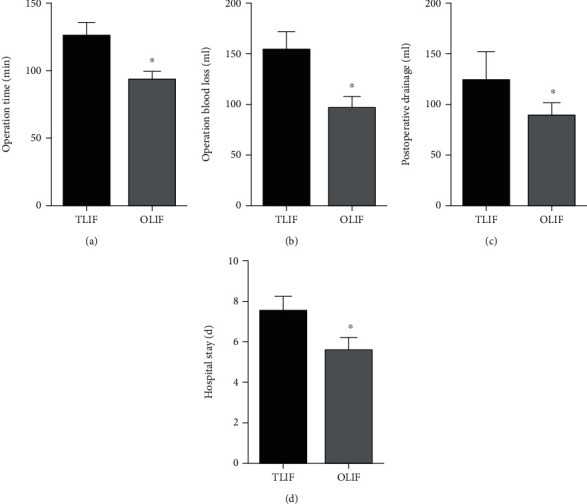
Comparison of operation time (a), operation blood loss (b), postoperative drainage (c), and hospital stay (d) between the two groups. (^∗^Compared with TLIF group, *P* < 0.05).

The OLIF group showed shorter operation time (*P* < 0.001), less operation blood loss (*P* < 0.001), less postoperative drainage (*P* < 0.001), and shorter hospital stay (*P* < 0.001) than the PLIF group. (Figure 2).

**Figure 3:**
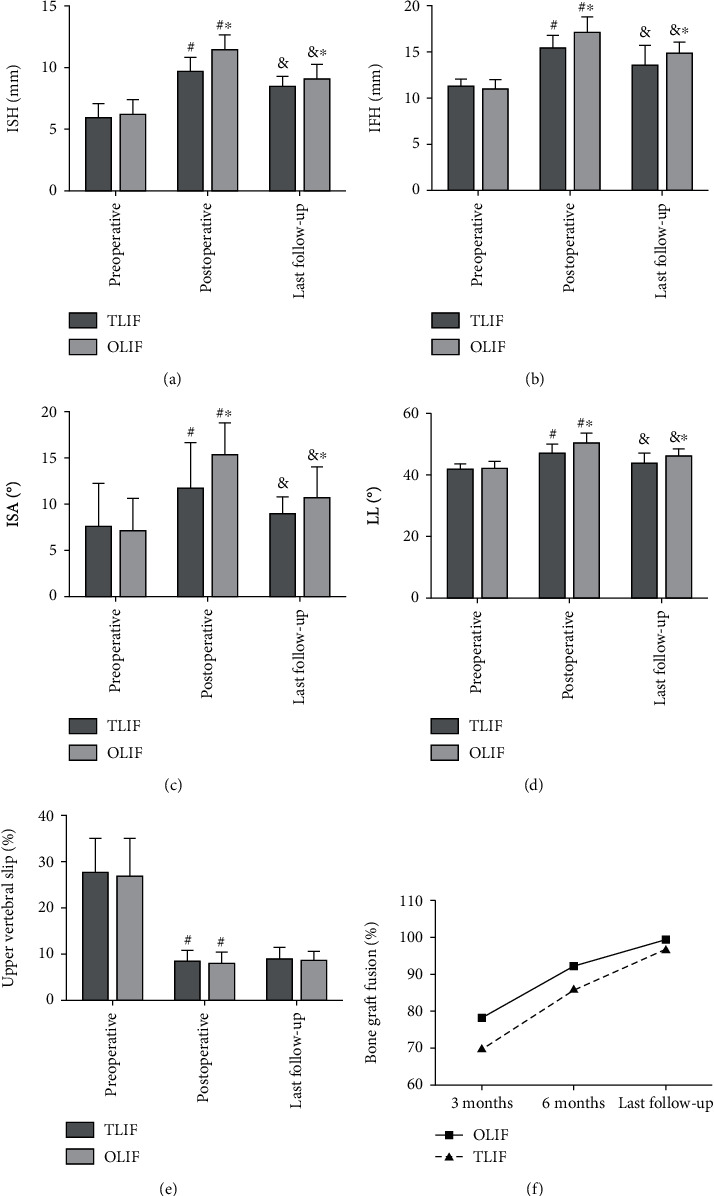
Comparison of ISH (a), ITH (b), ISA (c), LL (d), degree of upper vertebral slip (e), and bone graft fusion rate (f) between the two groups at different follow-up time. (^∗^Compared with TLIF group, *P* < 0.05; ^&^compared with preoperative, *P* < 0.05; ^#^compared with postoperative, *P* < 0.05).

No significant differences were found in preoperative ISH, IFH, ISA, and LL between the two groups (*P* = 0.508, 0.649, 0.231, and 0.522, respectively). However, the ISH, IFH, ISA, and LL at postoperative and the last follow-up were significantly larger in the OLIF group than those in the TLIF group (postoperative: *P* < 0.001, 0.002, <0.001, and<0.001, respectively; last follow-up: *P* = 0.032, 0.015, 0.014, and 0.002, respectively). The upper vertebral slip in the two groups was significantly corrected (*P* < 0.001 for both groups) and lost during the follow-up (*P* < 0.001 for both groups). But no significant differences were found in upper vertebral slip between the two groups at preoperative, postoperative, and last follow-up (*P* = 0.728, 0.453, and 0.531, respectively). The bone graft fusion rate of OLIF group and TLIF group for 3 months, 6 months, and last follow-up was 78.57%, 92.86%, and 100% and 70.27%, 86.49%, and 97.30%, respectively, and no differences were found between the two groups (*P* = 0.451, 0.412, and 0.389, respectively) (Figure 3).

**Figure 4:**
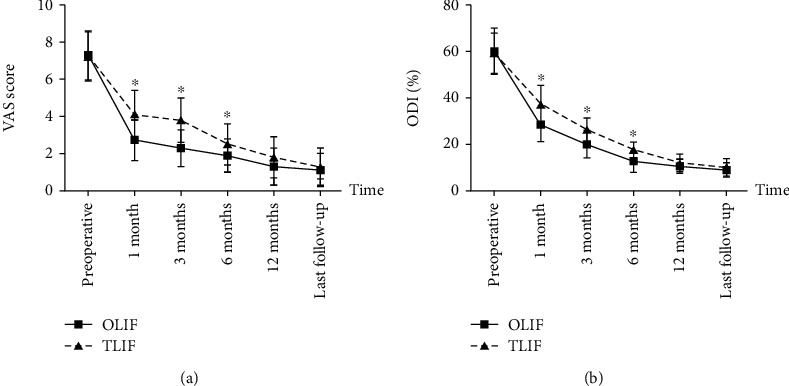
Comparison of VAS score (a) and ODI (b) between the two groups at different follow-up time. (^∗^Compared with TLIF group, *P* < 0.05).

No significant differences were found in VAS score and ODI between the two groups at preoperative, 12 months postoperative, and last follow-up (VAS score: *P* = 0.760, 0.064, and 0.408, respectively; ODI: *P* = 0.604, 0.088, and 0.216, respectively). However, the OLIF group showed a better improvement in VAS score and ODI at 1 month, 3 months, and 6 months postoperative than the TLIF group (VAS score: *P* < 0.001, *<*0.001, and 0.002, respectively; ODI: *P* < 0.001 for all the three, respectively) (Figure 4).

Four patients with complications were found in the OLIF group, including 2 patients of transient thigh flexion weakness, 1 patient of segmental artery injury, and 1 patient of transient sympathetic injury. While 6 patients with complications were found in the TLIF group, including 3 patients of dural tear, 1 patient of transient thigh pain and/or numbness, 1 patient of transient ankle dorsiflexion weakness, and 1 patient of postoperative incision infection. There was no significant difference in the complications rate (14.3% vs. 16.2%) between the two groups (*P* = 0.446). Finally, all complications were cured after treatment.

### 3.1. Typical Cases

**Figure 5:**
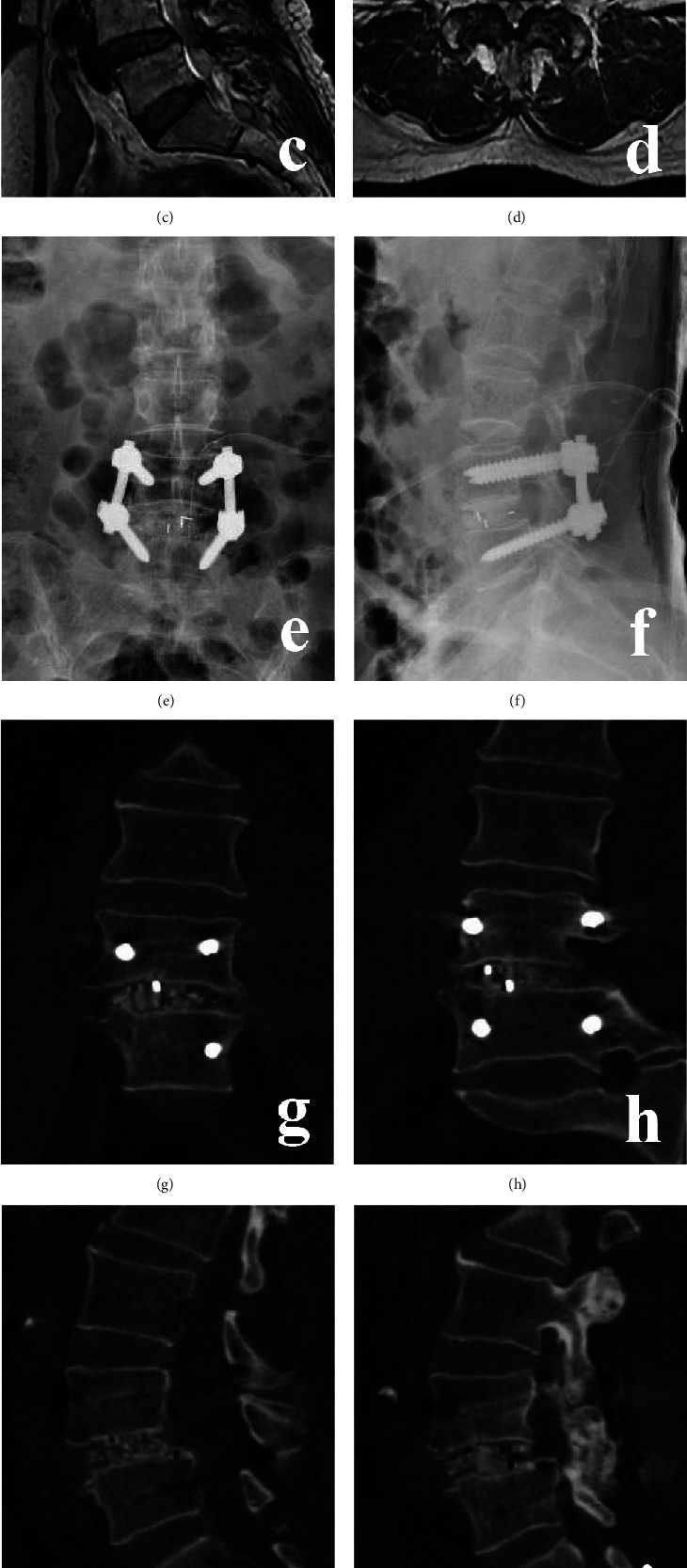
A 49-year-old male with L4/5 DLS in TLIF group. (a)–(d) Preoperative X-ray and MRI. (e) and (f) Postoperative X-ray. (g)–(j) CT at 6 months postoperative. (k) and (l) X-ray at 18 months postoperative.

**Figure 6:**
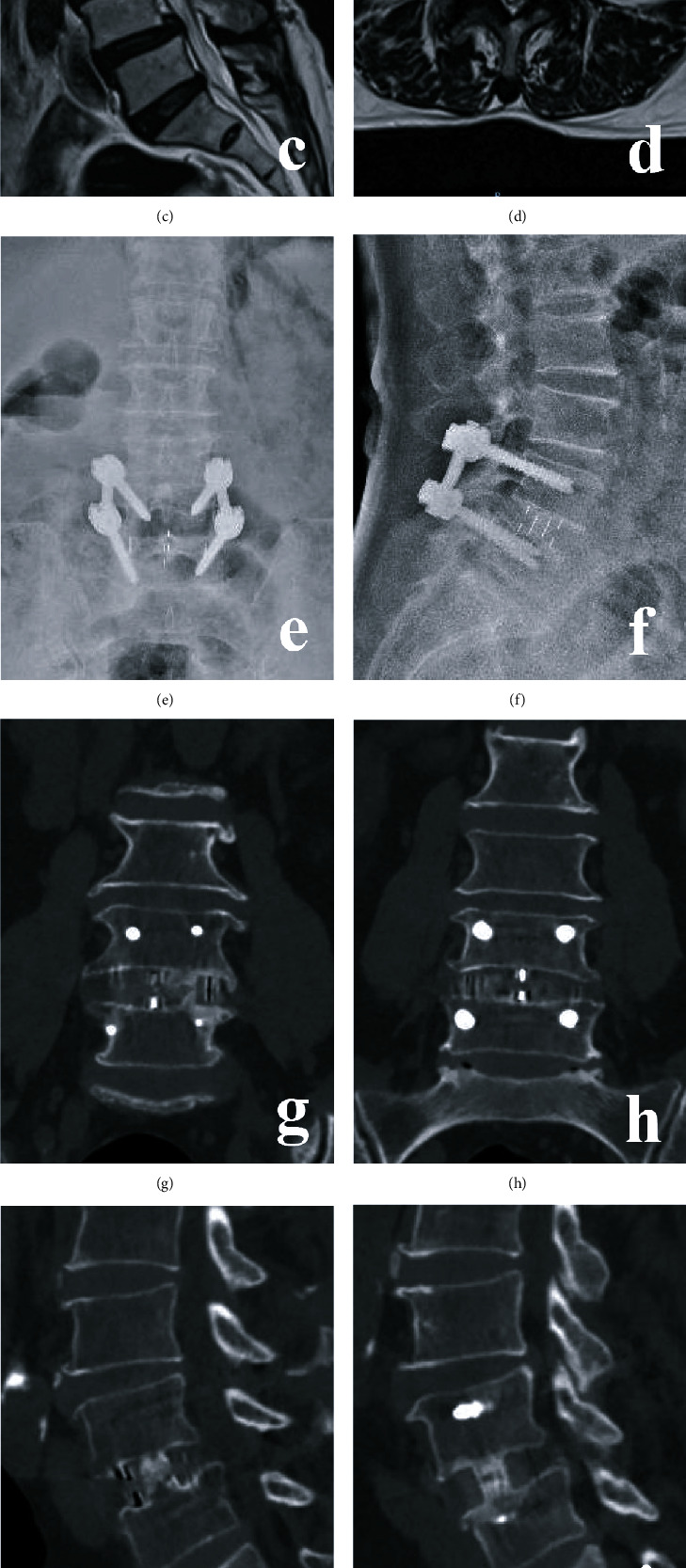
A 50-year-old female with L4/5 DLS in the OLIF group. (a)–(d) Preoperative X-ray and MRI. (e) and (f) Postoperative X-ray. (g)–(j) CT at 5 months postoperative. (k) and (l) X-ray at 20 months postoperative.

Typical cases were shown in Figures 5 and 6.

## 4. Discussion

In this study, TLIF showed longer operation time and more operation blood loss than OLIF. This result was similar to a previous study. The possible reasons were as follows: (1) in TLIF surgery, one side of paravertebral muscle was peeled off, and part of facet joints and lamina was also resected [17], while neither was done in OLIF surgery. (2) In OLIF surgery, surgeons could directly reach the operative intervertebral space and did the discectomy under direct vision [18], while in TLIF surgery, discectomy could not be performed under direct vision, especially for the contralateral side of the decompression side [19]. (3) In OLIF surgery, the spinal canal was not opened, so the risk of nerve injury was low, while TLIF surgery required opening the intervertebral foramina, which had a risk of nerve injury, so surgeons paid more attention to the separation and the protection of the nerve roots [20]. The less postoperative drainage and shorter hospital stay of the OLIF group may also be related to the larger surgical damage of TLIF surgery. In addition, the prolonged postoperative bedtime caused by the risk of spinal instability due to the intraoperative dissection of paravertebral muscle and facet joints may also be the reasons for the longer hospital stay of the TLIF group.

OLIF is an indirect decompression surgery, and many studies are aimed at evaluating the decompression effect of OLIF by comparing the changes of imaging parameters before and after surgery [21–23]. In our study, it was found that the ISH, IFH, and ISA for postoperative and last follow-up were significantly larger in the OLIF group than the TLIF group. The reasons may be as follows: (1) in OLIF surgery, a large cage with a degree of inclination angle was implanted into the intervertebral space, while in TLIF surgery, because of the narrow operating space, only a small cage, almost without an inclination angle, could be implanted through the intervertebral foramen [24]. (2) In TILF surgery, only one side of the paravertebral muscle and the facet joints was removed while the contralateral side was usually preserved, so the intervertebral space may not be effectively extended, especially for patients with severe facet joints degeneration or even facet joints fusion [25]. LL is an important imaging index to evaluate the efficacy of DLS surgery. It was confirmed that LL was closely related to postoperative lumbar back pain, and effective correction and maintenance of LL were of great significance to relieve lumbar back pain and improve lumbar function [26]. In this study, both the postoperative and last follow-up LL were found significantly larger in the OLIF group than the TLIF group. This was closely related to the effective correction and maintenance of ISH, IFH, and ISA in the OLIF group [27]. However, no differences in upper vertebral slip at preoperative, postoperative, and last follow-up were found between the two groups. This may be owing to the following reasons: (1) in TLIF surgery, the posterior pedicle system could effectively correct spondylolisthesis by its strong pulling force [28]. (2) Both of the two groups achieved a higher rate of bone graft fusion during the follow-up, and vertebral spondylolisthesis would not deteriorate once bone graft fusion was achieved. Although OLIF showed a better decompression efficacy than TLIF in this study, it is still necessary to extend the follow-up time to confirm this conclusion in future research, as spinal canal remodeling during long-term follow-up after surgery had been proved [29].

In the present study, the OLIF group and TLIF group showed similar bone graft fusion rate. We thought the reasons may be as follows: (1) in both groups, the intervertebral discs were completely removed, and the cartilage endplate was also completely scraped. This would create a suitable good graft bed for fusion [30]. (2) Granular bone graft was used in both groups, and our previous study had showed that granular bone graft had a high fusion rate [31]. (3) Both groups used a posterior pedicle screw system to create a stable local mechanical environment, which was also beneficial for bone graft fusion [32].

Postoperative pain relief and lumbar function recovery were the main focus of both the surgeons and patients. In this study, it was found that the short-term (in 12 months) postoperative pain and lumbar functional in the OLIF group were superior to the TLIF group, but there were no significant differences in long-term follow-up. The reasons may be as follows: (1) less damage to paraspinal muscles and facet joints was found in the OLIF group [33], so low back pain and lumbar function were better than the TLIF group in the short-term follow-up. (2) OLIF surgery did not open the spinal canal and had little stimulation to nerve roots [34]. (3) For most patients in both groups, bone graft fusion had achieved, and the paravertebral muscles had also been recovered at 1 year postoperative. The above results were also the reasons for shorter hospital stays in the OLIF group. Although stand-alone OLIF was reported with less injury to paravertebral muscles [33], PPSF was used in the OLIF group in our study, because preoperative BMD revealed that most patients had osteopenia or even osteoporosis.

Segmental artery injury, transient thigh numbness were the common complications in the OLIF group [35], because the lumbar plexus, lumbar sympathetic trunk, and segmental artery are all located laterally in front of the lumbar vertebrae and susceptible to being irritated or injured [36, 37]. The main complications of TLIF were nerve root stimulation or injury and dural tear [38]. However, we found no significant difference in complication rate between the two groups. This result also indicated the safety of the two surgical methods for DLS.

In our opinion, the indications of OLIF combined PPSF for DLS were as follows: (1) severe back pain and/or leg pain with poor response to regular conservative treatment of 3 months. (2) Progressive exacerbation of instability or spondylolisthesis. (3) Single-segmental DLS (L2/3-L4/5) with mild degree of spondylolisthesis (Grade I or II). (5) An appropriate operative window was shown between the psoas and abdominal aorta on preoperative MRI or CT [39].

This study also has some limitations. First, it is a retrospective study with a small sample size and short follow-up period. Second, different experiences in OLIF/TLIF surgery may also cause bias.

## 5. Conclusion

Compared with TLIF, OLIF showed the advantages of less surgical invasion, better decompression effect, and faster postoperative recovery in single-level DLS surgery.

## Figures and Tables

**Table 1:** Comparison of preoperative clinical features between the two groups.

Clinical features	TLIF group (*N* = 37)	OLIF group (*N* = 28)	*P* value
Age (year), mean ± SD	52.8 ± 7.1	53.6 ± 6.4	0.641
Gender (*n*), Male/Female	23/14	16/12	0.683
BMI (kg/m^2^), mean ± SD	22.5 ± 2.3	22.8 ± 2.1	0.591
ASA grade (*n*)			0.779
I	24	17	
II	10	9	
III	3	2	
Operation level (*n*)			0.890
L3/4	10	8	
L4/5	27	21	
BMD (*T* score), mean ± SD	−2.3 ± 1.0	−1.9 ± 0.9	0.101
Follow-up time (month), mean ± SD	22.1 ± 7.0	20.3 ± 6.1	0.282

## Data Availability

The clinical data in this study is available from the corresponding author on reasonable request.

## Abbreviations

DLS: Degenerative lumbar spondylolisthesis
ALIF: Anterior lumbar interbody fusion
TLIF: Transforaminal lumbar interbody fusion
OLIF: Oblique lateral interbody fusion
PPSF: Percutaneous pedicle screw fixation
VAS: Visual analog scale
ODI: Oswestry disability index
ISH: Intervertebral space height
IFH: Intervertebral foramen height
ISA: Intervertebral space angle
LL: Lumbar lordosis
SD: Standard deviation
BMI: Body mass index
BMD: Bone mineral density.

## Additional Points

*Research Registration*. This study had been registered in Chinese Clinical Trial Registry (Number: ChiCTR2000039446, http://www.chictr.org.cn/showproj.aspx?proj=63238).
